# Ulnar Neurectomy

**Published:** 1901-02

**Authors:** W. E. A. Wyman

**Affiliations:** Milwaukee, Wis.


### REPORTS OF CASES.

### ULNAR NEURECTOMY.

By W. E. A. Wyman, M.D.V., V.S.,
MILWAUKEE, WIS.

*A Course in Surgical Operations*, etc., by W. Pfeifer (Berlin), and translated by Prof. W. L. Williams, on page 67 describes ulnar neurectomy. About three years ago the writer had some interesting experience with this form of neurectomy, which may be of value to others.

The animal in this instance was a beautiful actor, and of that vicious type so prone to produce lameness, namely, by putting all vim into the start and slipping and sliding all over, thus acquiring a lameness in the right foreleg. The animal was brought to the writer with its shoulder setoned. Since the history of the case and the clinical symptoms were incompatible with shoulder lameness and distinctly those of carpitis, the diagnosis of carpitis was made. The writer suggested a thorough red iodide of mercury blister and prolonged rest. About five weeks had passed when the writer was again called upon to examine the horse. Once more a serious carpitis was diagnosed. Being desirous of convincing the owner that the horse was really knee lame, he insisting that it was rheumatism, I suggested a cocaine injection over the median nerve. This was done, and to my agreeable surprise the animal trotted around sound for about one-half hour—I say to my agreeable surprise, simply for the reason that I was not certain about locating the median nerve in this decidedly unruly creature. A blister was at once applied. Six weeks later a little exercise still showed the animal lame. Now, the animal was puncture fired and blistered, which treatment was repeated at the end of the sixth week, as the horse was still lame. Six more weeks, and innumerable discussions as to the eventual outcome of the case, taxing the patience of a saint, passed by, and the horse remained lame. What next? The owner looked upon neurectomy as a murder. The horse, therefore, was returned to pasture. The fact that the owner had to pay board regularly for the horse, which yielded no returns, softened his heart toward neurectomies; then I was requested to lay down my views regarding neurectomies before him and a formidable array of so-called expert horsemen. After a decided waste of breath and time the writer was permitted to perform mesoneurectomy. At the end of the third week the animal was exercised lightly, and, while decidedly improved, nevertheless exhibited some lameness, showing that the diseased area extended over a greater space than that controlled by the median nerve. What next? My illustrious friend and teacher, Dr. M. H. McKillip, has a great standard: "Always have something else up your sleeve." I admit I shook my mental sleeve until the buccal alveoli threatened to become empty, when suddenly it occurred to me that the removal of a bit of the ulnar nerve might solve the question. This was done, and the wound healed by first intention. Oh, the intentions were good, and in due time the horse was returned to the owner without lameness. He used the animal every day for about four weeks, at which time the animal was sold and went to a different State.

After a stay at the new owner's home for two weeks the groom was much perplexed to find the horse, one morning, with the toe of the right forefoot turned up into the air—rupture of the perforans, possibly explained by the sudden change of climate (?).

Mesoneurectomy has been employed by the writer a great many times without any such accidents up to date; therefore, the writer bought four horses (no high actors or high-priced) which, as far as detectable, were sound in front. These received ulnar neurectomy and were given to a young man who used them on his farm. All four were killed not later than six months after the operation of ulnar neurectomy, on account of rupture of the perforans tendon. Ulnar neurectomy I have since discarded. These cases remind me very much of a story I heard in Germany, and, while not connected with neurectomies, nevertheless may have some effect upon the nervatory apparatus of man, since it conveys the moral of the above to a nicety.

There was once—many years ago—a barber, who, during moments of leisure, practised his empiric knowledge of medicine upon the credulous ones of his district. He had many admirers, and among them two unfortunate ones—one a blacksmith, the other a tailor. This contingent of two, according to the able diagnosis made by the barber-doctor, were afflicted with consumption. The blacksmith was still able to be about, while the tailor was bed-ridden and about to die. The story goes on and reports that one day a stranger came to the tonsorial artist and offered a sure cure for consumption. Here was the chance of his life. The stranger received the fifty francs he asked for the formula, the barber entering it as No. 52. (In those days irrigations of the trachea and bronchi were unknown.)

In a few days the preparation was ready for dispensation, and, armed with his specific, the barber proceeded to visit the tailor and blacksmith, informing both of the sacrifice he had made in their behalf, assuring them now of a speedy recovery. The barber-doctor's annotation in regard to prescription No. 52 was: "This medicine cures consumption in blacksmiths but kills tailors." The moral: Ulnar neurectomy removes lameness but ruins the horse. While criticising I may also include in this the operation for roaring —"arytenoideraphy"—as, to say the least, an unreliable operative measure, for reasons to be exhibited another time.

---

Dr. Wardle, a well-known young veterinary surgeon and sportsman, of Montreal, Canada, died rather suddenly at the age of thirty-eight years. He was the owner of several well-known steeplechasers, among them "Bushbolt," a winner of the Herald Cup at Belair track; "Felix," which he purchased from J. E. Seagram, M. P., of Waterloo, Ont., and "Billy McKinley." These three animals were killed at the request of their late owner, Dr. Wardle's will containing this clause:

"It is my wish and desire that a week after my death my three horses, 'Billy McKinley,' 'Bushbolt,' and 'Felix,' shall be put to death in the most painless manner possible. This is my desire, because I would be much grieved should the animals, after the affection and care I have had for them, fall into the hands of people who would treat them cruelly or make them work or do anything which would make them suffer."

Confronted by this unusual clause in the will, the executors had no alternative but to obey, and so the three valuable animals were taken out to the club-house of the Montreal Hunt Club, behind Mount Royal, and there killed, each in his turn, by a shot from a revolver in the hands of Dr. Ernest Thurston. It is said that the horse "Billy McKinley" refused to eat from the time he missed his late master.

Feeding experiments of sheep with different rations to test their value as meat-producing foods are being conducted at the University of Nebraska.

